# The Role of T Follicular Helper Cells and Interleukin-21 in the Pathogenesis of Inflammatory Bowel Disease

**DOI:** 10.1155/2021/9621738

**Published:** 2021-08-23

**Authors:** Lulu Sun, Ruixue Kong, Hua Li, Dashan Wang

**Affiliations:** ^1^Shandong Provincial Key Laboratory of Animal Resistance Biology, College of Life Sciences, Shandong Normal University, Jinan, Shandong 250014, China; ^2^Research Center, Shandong Medical College, Linyi, Shandong 276000, China

## Abstract

T follicular helper (Tfh) cells represent a novel subset of CD4^+^ T cells which can provide critical help for germinal center (GC) formation and antibody production. The Tfh cells are characterized by the expression of CXC chemokine receptor 5 (CXCR5), programmed death 1 (PD-1), inducible costimulatory molecule (ICOS), B cell lymphoma 6 (BCL-6), and the secretion of interleukin-21 (IL-21). Given the important role of Tfh cells in B cell activation and high-affinity antibody production, Tfh cells are involved in the pathogenesis of many human diseases. Inflammatory bowel disease (IBD) is a group of chronic inflammatory diseases characterized by symptoms such as diarrhea, abdominal pain, and weight loss. Ulcerative colitis (UC) and Crohn's disease (CD) are the most studied types of IBD. Dysregulated mucosal immune response plays an important role in the pathogenesis of IBD. In recent years, many studies have identified the critical role of Tfh cells and IL-21 in the pathogenic process IBD. In this paper, we will discuss the role of Tfh cells and IL-21 in IBD pathogenesis.

## 1. Introduction

T follicular helper (Tfh) cells are a specialized subset of T helper (Th) cells that can migrate into germinal center (GC) and help B cells to differentiate into antibody-producing plasma B cells and generate high-affinity antibodies [1]. In GC, Tfh cells provide survival and differentiation signals to B cells by cell-surface molecule crosstalk and the secretion of interleukin-21 (IL-21) [2]. Tfh cells play a central role in B cell activation and high-affinity antibody production; however, the dysfunction of Tfh may lead to allergic reactions, systemic autoimmune diseases, and chronic inflammation [3, 4].

Inflammatory bowel disease (IBD) is a group of chronic inflammatory disorders characterized by chronic relapsing inflammation of the gastrointestinal tract. Ulcerative colitis (UC) and Crohn's disease (CD) are the major forms of inflammatory bowel disease (IBD) [5–7]. Although the exact etiology of IBD remains unclear, it is well established that genetic factors, environmental factors, microbiota, and immune response all contribute to this disease. Among them, the role of immune response in the development of IBD attracts many interests. In the gut of these patients, the tissue-damaging immune response is initiated and regulated by the interplay between the immune and nonimmune cells [8–10]. Many studies have revealed that immune cells such as Th1, Th2, Treg, and Th17 contribute to the pathogenesis of IBD [11, 12]. In recent years, more and more studies have discovered the critical role of Tfh and IL-21 in initiating and shaping the pathologic process of IBD. In this article, we will discuss the pathogenic role of Tfh cells and IL-21 in IBD.

## 2. T Follicular Helper (Tfh) Cell Differentiation

B lymphocytes and T lymphocytes are two important cell populations in the adaptive immune system. T cell-mediated cellular immunity and B cell-mediated humoral immunity are two types of adoptive immunity. The generation of neutralizing antibodies by B lymphocytes is a critical step in immune response to viral or bacterial infections, which is one of the two types of adoptive immunity. This process needs direct crosstalk between activated B and T cells in a specialized structure called germinal center (GC). The germinal center (GC) formation is critical in the generation of high-affinity antibodies and long-lived plasma cells, which is the base of humoral immune responses against pathogen infection. CD4^+^ helper T cells have been found to play critical roles in this progress [13–15]. In early 2000s, a new subset of CD4^+^ helper T cells termed as T follicular helper (Tfh) cells have been identified as the key cells necessary for regulating GC formation and B cell function. Tfh cells are the key mediator in regulating humoral immune response via direct interactions with B cells [16–18]. The Tfh cells have many unique features, such as the expression of CXC chemokine receptor 5 (CXCR5), programmed death 1 (PD-1), inducible costimulatory molecule (ICOS), and B cell lymphoma 6 (BCL-6) and the production of IL-21 [19–21].

**Figure 1:**
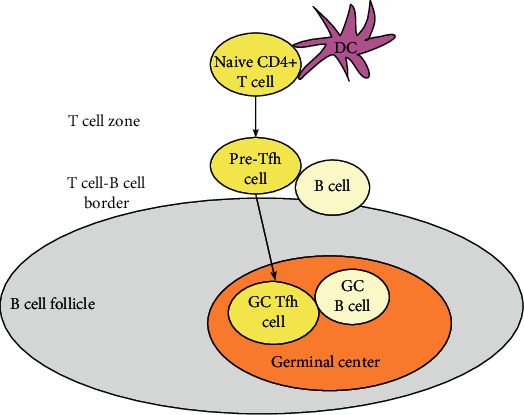
The differentiation of Tfh cells. Naive CD4^+^ T cells are primed by MHC/antigen interaction with DCs and differentiate to pre-Tfh cells; then, pre-Tfh cells migrate to the T-B border and interact with cognate B cells; finally, interactions between pre-Tfh cells and B cells lead to the formation of GC Tfh cells.

Tfh cell differentiation is a complex and multistage process (Figure 1). Initially, naive CD4^+^ T cell are stimulated by DCs in a T cell zone of secondary lymphoid tissues and become to pre-Tfh; then, guided by chemokines, pre-Tfh migrates to the T-B border and interacts with antigen-specific B cells in B cell follicles; further stimulation and antigen presentation by B cells provide help to pre-Tfh cells to become GC Tfh cells. GC Tfh cells can help B cells to differentiate into antibody-secreting plasma cells in GCs [22–24]. Tfh cell differentiation is regulated by various factors such as extracellular cytokines, cell-surface molecule interactions, multiple transcription factors, and microRNAs [25, 26].

### 2.1. Cytokines

#### 2.1.1. IL-6

Cytokine signaling play a critical role in driving naive CD4^+^ T cells to differentiate into distinct effector T cell subsets. Numerous studies have reported the important role IL-6 in regulating Tfh cell differentiation [27]. IL-6 can promote Bcl-6 expression and Tfh cell differentiation by regulating signal transducer and activator of transcription 1 (STAT1) and STAT3 signaling pathway [28]. The deficiency of IL-6 led to delayed Tfh cell formation [29].

#### 2.1.2. IL-21

IL-21 has many regulatory effects on innate and adaptive immune responses [30]. IL-21 can drive Tfh cell differentiation via the STAT3 signaling pathway. IL-21 deficiency in mice resulted in a decreased number of Tfh cells after protein immunization [18]. Besides, Tfh cells can produce IL-21, and IL-21 was necessary for GC formation [31]. IL-21 was also critical in regulation of human B cell differentiation into Ig-secreting cells [32].

### 2.2. Cell-Surface Factors

#### 2.2.1. CXCR5

CXC chemokine receptor 5 (CXCR5) is an important Tfh cell surface marker. CXCR5 is responsible for the migration of T cells to the B cell zone and thereby lead to T : B cell interaction. By upregulation CXCR5 and downregulation of CCR7, these CD4^+^ T cells migrate to B cell follicles where CXCL13 is expressed, interact with B cells for further maturation, and provide help for B cell activation and GC reactions [33, 34]. CXCR5 can also act as an important factor in modulating Tfh cell differentiation. Knockout of CXCR5 resulted in decreased GC number and humoral immune response [35].

#### 2.2.2. CD28

Cell-surface costimulators, which are important for T cell-B cell interaction, are also responsible for Tfh cell development. The CD28-B7 costimulatory signal pathway is critical for Tfh cell differentiation and GC reaction [36, 37]. CD28 signaling positively regulates the differentiation and survival of Tfh cells. The deficiency of CD28 in mice led to absence of Tfh cells and blocking of CD28 signal inhibited expression of Bcl-6 [38].

#### 2.2.3. ICOS

Inducible costimulator (ICOS) is another costimulator which can bind to ICOSL. ICOS is expressed on Tfh cells and is responsible for Tfh cell differentiation. *ICOS^–/–^* and *ICOSL^–/–^* mice exhibited impaired Tfh generation cell and GC formation. Tfh cell generation and GC formation were significantly impaired in ICOS-deficient patients [39]. ICOS was essential to maintain the phenotype of Tfh cells by regulating transcription factor Klf2 [40]. It was reported that the ICOS-PI3K signaling pathway is critical for Tfh differentiation. The ICOS-PI3K signaling pathway resulted in enhanced Bcl-6 expression and Tfh cell differentiation [41].

#### 2.2.4. PD-1

Programmed death 1 (PD-1), a cell surface factor expressed on exhausted T cells, is also expressed on Tfh cells. PD-1 was initially expected to be a negative regulator in Tfh cell differentiation. Mice with impaired PD-1 signaling showed more Tfh cell numbers after protein immunization [42]. PD-L1-PD-1 interaction controls tissue positioning and function of Tfh cells [43]. PD-L1 can also inhibit T follicular regulatory cells (Tfr) differentiation; the number of Tfr is increased in the absence of PD-1 signaling. PD-1 is expressed by Tfr and also involved in regulating Tfr cells functions [44].

### 2.3. Transcription Factors

#### 2.3.1. Bcl-6

It is well established that Tfh cells comprise a distinct subset of Th cells and possess a unique gene expression profile. A breakthrough discovery in Tfh cells research is the identification of the master transcription factor, B cell lymphoma 6 (Bcl-6). Bcl-6 plays an essential role in development and function of Tfh cells. Tfh cells cannot be generated in the absence of Bcl-6, whereas overexpression of Bcl-6 could restore this defective Tfh cell differentiation [45, 46]. It was reported that Bcl-6 promoted expression of Tfh marks like CXCR5 and PD-1; however, it repressed the expression of genes critical for other T helper lineages, such as T-bet, IFN-*γ*, ROR-*γ*t, and IL-17. Bcl-6 acted as a repressive transcription factor which can bind to the promoter of *Tbx21* and *Rorc*, encoding T-bet and RoR-*γ*t, and inhibit development of Th1 and Th17 cells, respectively [47].

#### 2.3.2. STATs

Signal transducer and activator of transcription (STAT) pathways have a positive or negative role in Tfh cell differentiation. STAT1, STAT3, and STAT4 can promote Tfh cell development. The deficiency of these STATs led to the reduction of Tfh cells [48–50]. In contrast, STAT5 plays a negative role in Tfh cell development and humoral immunity. Inhibition of the STAT5 signaling pathway resulted in enhanced Bcl-6 expression, Tfh cell differentiation, and GC formation [51, 52].

#### 2.3.3. Blimp-1

B lymphocyte-induced maturation protein 1 (Blimp-1), a transcriptional factor in regulating of terminal B cell differentiation and function, also plays a role in Tfh cell differentiation. Blimp-1 is an antagonist of Bcl-6; thus, Blimp-1 can inhibit Bcl-6 expression and repress Tfh differentiation [53, 54].

### 2.4. MicroRNAs

Studies also identified the critical role of microRNAs in Tfh cell differentiation. miR-17 approximately 92 family acts as an important factor in Tfh differentiation during viral infection or protein immunization [55]. miR-155 promoted Tfh cell formation and contributes to development of inflammatory disease during chronic low-grade inflammation [56]. However, miR-146a repressed Tfh cell differentiation by regulating the ICOS/ICOSL signaling pathway [57].

## 3. Inflammatory Bowel Disease

Inflammatory bowel disease (IBD) is a chronic inflammatory disorder characterized by symptoms such as diarrhea, abdominal pain, and weight loss. Ulcerative colitis (UC) and Crohn's disease (CD) are the two major subtypes of IBD [58]. IBD is a worldwide health problem with a continually increasing incidence. Although the exact etiology is still not fully understood, it is well-known that personal genetic susceptibility, environment, intestinal microbiota, and immune system all contribute to the pathogenesis of IBD. Among them, deregulation of immune response in the intestinal mucosa acts as a master factor in the etiology of IBD [59]. The local immunity in the intestinal mucosa is critical for microenvironment homeostasis, as intestinal immune system not only maintains protective immunity against invading pathogens but also controls immune tolerance to commensal microbiota. Nevertheless, the dysregulated immune response against self-antigens or commensal microbiota can lead to mucosal inflammation. Immune cells such as macrophages, neutrophils, dendritic cells, T cells, and B cells have been proved having critical roles in regulating mucosal inflammation [60, 61].

## 4. The Role of Tfh Cells and IL-21 in IBD

The major function of Tfh cells is to help B cells to generate neutralizing antibodies against pathogenic infection, wherein the dysregulated Tfh cell function is also involved in a range of disorders. Tfh cells can perform their functions through IL-21 [62]. In recent years, increasing evidence has implicated that Tfh cells and the related cytokine IL-21 participate in the pathogenesis of IBD.

### 4.1. The Role of Tfh Cells and IL-21 in Ulcerative Colitis

#### 4.1.1. Tfh Cells in Ulcerative Colitis

Ulcerative colitis (UC), one of the most frequent forms of IBD, is characterized by mucosal inflammation limited to the colon. The clinical symptoms of UC include blood and mucus release, granulation tissue, and petechial hemorrhage [63]. Recently, several studies have reported that Tfh cells have a role in the pathogenesis of UC.

Tfh cells were elevated in UC patients [64–68]. The value of Mayo clinic score, erythrocyte sedimentation rate (ESR), or C-reactive protein (CRP) in UC patients was positively correlated with frequency of circulating Tfh cells [64]. Bcl-6 mRNA and Tfh cells in the germinal center were increased in the UC groups compared with that in the control group [65]. Recent studies further showed that Tfh cells were significantly increased in active UC patients compared with stable remission UC patients and healthy controls. The level of Tfh cells was positively correlated with circulating new memory B cells, plasmablasts, serum IgG, IL-4, IL-21, serum CRP, and Mayo scores [66]. The level of Tfh cells was significantly decreased when active UC patients achieve clinical remission after treatment. Tfh cells can be used as a biomarker in distinguishing active UC from stable remission patients [67]. Another research also found that the number of Tfh cells was significantly increased in colonic tissues from UC patients [68]. These results implicated that Tfh cells are critical regulators in colitis development.

Activated B cells can highly express the CD86, and the CD86^+^ B cells can differentiate into CD38^+^ plasma B cells which can produce antibodies. Dysregulated B cell response is associated with the pathogenesis of IBD [69, 70]. One study found that the percentages of circulating activated B cells (CD86^+^CD19^+^) and plasma B cells (CD38^+^CD19^+^) were both significantly higher in UC patients than in healthy controls. The percentages of activated B cells, plasma B cells, and serum IgG concentration were positively correlated the percentage of Tfh cells. Tfh cells might regulate the development of UC by controlling the B cell function [71].

Tfh cells can also participate in pathogenesis of UC via a B cell-independent manner. One research showed that adoptive transfer of naive CD4^+^ T cells to *Rag1^−/−^* recipients will lead to development of colitis; however, adoptive transfer of naive *Bcl6^−/−^* CD4^+^ T cells into Rag1^−/−^ recipient cannot induce Tfh cell differentiation and the development of colitis [72].

T follicular regulatory cells (Tfr) are a specific regulatory T cell subgroup expressing CXCR5 and FoxP3. Tfr can inhibit Tfh cell function and thus suppress humoral immunity [73]. The suppression function of Tfr cells was significantly lower in UC patients than in healthy controls [74]. UC patients exhibited significant reductions in circulating Tfr cells. The value of the Mayo clinic score, erythrocyte sedimentation rate (ESR), or C-reactive protein (CRP) in UC patients was negatively correlated with circulating Tfr cells and Tfr/Tfh ratio [64]. The number of Tfr cells was also reduced in the intestinal germinal center of the UC groups compared with that of the control group [65]. Recent studies further showed that circulating Tfr was decreased in active UC patients compared with stable remission UC patients and healthy controls and Tfr was negatively correlated with serum IgG, circulating new memory B cells, plasmablasts, and clinical characteristics [66].

#### 4.1.2. IL-21 in Ulcerative Colitis

The level of IL-21 is elevated in UC patients compared to healthy controls [64, 65, 71, 75]. Serum IL-21 was significantly higher in active UC compared with HC and stable remission UC patients [66]. The percentage of CD3^+^CD8^−^IL-21^+^ T cells in peripheral whole blood was significantly elevated in UC patients [76]. There is also a study that reported that the percentage of IL-21-producing CD4^+^ T intestinal lamina propria lymphocytes (T-LPL) was increased in IBD compared with controls [77]. There is a positive correlation between Tfh cells and the level of IL-21 in UC patients [66, 71]. Besides, the value of Mayo clinic score, erythrocyte sedimentation rate (ESR), or C-reactive protein (CRP) in UC patients was positively correlated with serum IL-21 concentration [64]. IL-21 knockout (IL-21KO) mice were resistant to dextran sulphate sodium- (DSS-) induced colitis. Moreover the count of infiltrating Tfh cells and secretion of Tfh-related cytokines were significantly decreased in IL-21KO mice in comparing to WT mice. Blockade of IL-21 by administration of a neutralizing IL-21 antibody decreased the colonic infiltration of Tfh cells and inhibited development of DSS-induced colitis [68]. Anti-IL-21 mAb treatment also ameliorated CD4^+^CD25^−^ T cell adoptive transfer (AdTr) Colitis [78].

IL-21 might be available for tight monitoring of the disease activity of UC. Serum IL-21 was significantly higher in UC patients with unstable remission compared to UC patients with stable remission. Serum IL-21 was also elevated in UC patients with later occurring relapses [79]. Another research also found that a higher IL-21 level might be an increased risk of UC relapse [80].

### 4.2. The Role of Tfh Cells and IL-21 in Crohn's Disease

#### 4.2.1. Tfh Cells in Crohn's Disease

Crohn's disease (CD) is another form of IBD. Different from UC, the inflammation in CD can occur throughout the entire gastrointestinal tract. The clinical manifestations of CD are characterized by abdominal pain, diarrhea, malnutrition, and weight loss [81]. In recent years, studies have proved the important role of Tfh cells in pathogenesis of CD.

The percentage of circulating Tfh cells in peripheral blood was significantly elevated in CD patients than in controls, and the percentage of circulating Tfh was significantly increased in penetrating CD compared with inflammatory CD or stricturing CD [82]. The number of Tfh cells was increased, and the number of Tfr cells was decreased in the intestinal germinal center of the CD groups compared with that of the control group [65]. Another study also reported that the percentage of IL-21-positive Tfh cells was higher in CD patients than in controls [77]. Tfh cell frequencies in the intestine and peripheral blood were associated with CD activity. CD patients with clinical response to ustekinumab (IL-12 and IL-23 antagonists) had reduced frequencies of circulating Tfh cells in a concentration-dependent manner. Ustekinumab inhibited Tfh cell differentiation *in vitro*, and ustekinumab therapy can reduce the germinal center activity in CD patients *in vivo* [83].

#### 4.2.2. IL-21 in Crohn's Disease

IL-21 and IL-21R were observed in the mucosa and submucosa from healthy controls within normal limits; however, in patients with CD, IL-21 and IL-21R were additional expressed in lymphoid aggregates of muscularis externa. IL-21R was significantly highly expressed in the intestine from patients with CD compared with that in controls [78]. The expression of IL-21 was upregulated in the CD group compared with that in the control group [65, 75, 79].

## 5. Conclusion

Tfh cells are a novel subset of CD4 T cells which can provide critical help for germinal center (GC) formation and antibody maturation. Because of the critical role of Tfh cells in adaptive immune response, the accurately control of their differentiation and function is very important. There is increasing evidence that dysregulation of Tfh activities is implicated in the pathogenesis of many autoimmune diseases including IBD. Tfh cells and Tfh-related molecules may become a novel therapeutic target in treatment of IBD in future.
